# Receptor, Ligand and Transducer Contributions to Dopamine D2 Receptor Functional Selectivity

**DOI:** 10.1371/journal.pone.0141637

**Published:** 2015-10-30

**Authors:** Sean M. Peterson, Thomas F. Pack, Marc G. Caron

**Affiliations:** 1 Department of Cell Biology, Duke University Medical Center, Durham, North Carolina, United States of America; 2 Department of Neurobiology, Duke University Medical Center, Durham, North Carolina, United States of America; 3 Department of Medicine, Duke University Medical Center, Durham, North Carolina, United States of America; Indian Institute of Technology Kanpur, INDIA

## Abstract

Functional selectivity (or biased agonism) is a property exhibited by some G protein-coupled receptor (GPCR) ligands, which results in the modulation of a subset of a receptor’s signaling capabilities and more precise control over complex biological processes. The dopamine D2 receptor (D_2_R) exhibits pleiotropic responses to the biogenic amine dopamine (DA) to mediate complex central nervous system functions through activation of G proteins and β-arrestins. D_2_R is a prominent therapeutic target for psychological and neurological disorders in which DA biology is dysregulated and targeting D_2_R with functionally selective drugs could provide a means by which pharmacotherapies could be developed. However, factors that determine GPCR functional selectivity *in vivo* may be multiple with receptors, ligands and transducers contributing to the process. We have recently described a mutagenesis approach to engineer biased D_2_R mutants in which G protein-dependent (^[Gprot]^D_2_R) and β-arrestin-dependent signaling (^[βarr]^D_2_R) were successfully separated (Peterson, *et al*. PNAS, 2015). Here, permutations of these mutants were used to identify critical determinants of the D_2_R signaling complex that impart signaling bias in response to the natural or synthetic ligands. Critical residues identified in generating ^[Gprot]^D_2_R and ^[βarr]^D_2_R conferred control of partial agonism at G protein and/or β-arrestin activity. Another set of mutations that result in G protein bias was identified that demonstrated that full agonists can impart unique activation patterns, and provided further credence to the concept of ligand texture. Finally, the contributions and interplay between different transducers indicated that G proteins are not aberrantly activated, and that receptor kinase and β-arrestin activities are inextricably linked. These data provide a thorough elucidation of the feasibility and malleability of D_2_R functional selectivity and point to means by which novel *in vivo* therapies could be modeled.

## Introduction

G protein-coupled receptors (GPCRs) are dynamic conduits of extracellular messages into complex intracellular instructions. These instructions are carried out through activation of both G protein dependent and independent signaling pathways [1]. GPCRs exhibit *functional selectivity* [2] in responses to natural or synthetic ligands by signaling through a subset of their normal multiple pathways. This capacity for functional selectivity has been theorized to arise from receptor conformational heterogeneity. Additionally, ligands that exhibit functional selectivity are termed *biased agonists* [3]. The concept of functional selectivity at GPCRs could provide a means not only to understand how GPCRs mediate their actions but also for developing more selective and efficacious therapeutic agents. Based on this concept an increasing number of functionally selective or biased ligands have been developed for a several GPCRs [4–12]. Most GPCRs, with the exception of a few [13,14], mediate the action of a single natural ligand. How *in vivo* pleiotropic signaling is determined following engagement of the receptor by its cognate ligand is likely to be controlled by the complement of cellular accessory proteins, such as G proteins, GPCR kinases (GRKs) and β-arrestins.

One of the major transducers of G protein independent signaling are β-arrestins, multifunctional adaptor proteins and part of the desensitization machinery that scaffold GPCRs for internalization and recycling of competent receptors to the plasma membrane [15]. β-arrestins also scaffold signaling complexes that have been shown to alter metabolic pathways [16], transcription [17], and neuronal activity leading to behavior [18]. For those GPCRs for which the consequences of functionally selective signaling have been examined, the G protein and β-arrestin signaling pathways typically subserve different cellular functions [19–21] but with some notable exceptions, as observed with the AT1A receptor in its regulation of aldosterone production [22]. The molecular details of selectivity and major signal transduction elements of β-arrestin or G protein signaling are new avenues by which GPCR pharmacology can be exploited for the development of novel pharmaceutical therapies [23].

The dopamine D2 receptor (D_2_R) is a prominently expressed GPCR for the biogenic amine dopamine (DA). DA is critical in many central nervous system functions and D_2_R is the target of many pharmaceutical interventions in which DA homeostasis is disrupted. D_2_R responds to DA with activation of the inhibitory family of G_αO/i_ subunits which leads to an inhibition of cAMP production and liberation of G_βγ_ which leads to MAP kinase activation, as well as increased cell membrane potassium conductance through GIRK channels, among other effects [24]. Additionally, genetic and biochemical approaches have implicated β-arrestin 2 as a significant contributor to D_2_R signal transduction [18].

Functional selectivity arises from receptor conformational heterogeneity, which is the receptor’s capacity to adopt multiple related conformations that activate signaling molecules [25,26]. GPCRs undergo two major conformational processes during activation: 1) G protein stimulation and 2) β-arrestin recruitment. Ligands stabilize the transition state for guanine-nucleotide exchange factor (GEF) activity of GPCRs at G proteins, while GPCR kinases (GRKs) are efficiently recruited to agonist bound GPCRs where they phosphorylate intracellular domains, most frequently the C-terminal tail of GPCRs. Phosphorylation alters the agonist bound receptor conformational ensemble to favor β-arrestin recruitment, and this presumably initiates G protein-independent signaling.

Understanding how GPCRs propagate pleiotropic signals to generate functionally selective responses depends on the question at hand. In developing a selective ligand, recognition of receptor conformational states by a ligand may guide the experimental approaches. However, if an altered signaling mechanism mediating a specific cellular effect is desired for therapeutic benefit, distinct determinants of the selectivity process may be invoked. An important caveat in the design of biased agonists is the relative expression levels of transducer/interacting molecules, which can determine the bias because of altered coupling probability [27]. D_2_R’s prominence as a pharmaceutical target for many disorders makes it a good receptor candidate for precise and robust dissection of functional selectivity. We have recently reported the development and characterization of mutant D_2_R that are selective for G protein activation or β-arrestin recruitment [28]. These mutants, termed ^[Gprot]^D_2_R and ^[βarr]^D_2_R, respectively, show an unprecedented separation of function and have retained essentially the unabridged major functions of ^[WT]^D_2_R.

Here, the contributions of ligand, receptor and transducer to functional selectivity are systematically assessed using several variants of the previously described mutants. These novel mutants are characterized and assessed for their unique functional selectivity properties that provide insight into the quality and determinants of D_2_R functional selectivity. The data demonstrate that functional selectivity is dynamic and malleable. In addition to direct manipulation of the receptor, the role of transducer levels altered the signaling profile of D_2_R, which suggests that the *in vivo* complement and expression level of transducers play a significant role in shaping the functional selectivity of D_2_R ligands.

## Materials and Methods

### Mutagenesis PCR

The Agilent Technologies (Santa Clara, CA) QuikChange mutagenesis kit was used to carry out all mutagenesis according to manufacturer’s instructions. Primers were designed as instructed, with the minimum amount of nucleotide changes required to achieve a mutation. All work was carried out on the mouse long isoform of D_2_R. Multiple point mutations were created by using the same primers for single point mutations on already mutated constructs. All constructs were confirmed to have no coding errors by sequencing. In addition, a previously characterized D_2_R mutant, termed ^[D80A]^D_2_R which was previously shown to ablate sodium coordination which causes a deficit in G protein [29] and β-arrestin recruitment [28] but not ligand binding or plasma membrane trafficking was used as a negative control.

### Cell culture and transfections

HEK-293T (ATCC, Manassas, VA) cells were cultured and transfected as previously reported [30].

### G protein activity

D_2_R’s ability to inhibit cAMP production was carried out as previously described [8] using the Promega (Madison, WI) GloSensor assay with minor modifications. D_2_R was expressed at a mass of 1 μg of DNA (except where indicated) and the GloSensor construct was transiently transfected along with D_2_R at a mass of 5 μg of DNA. The luminescence was quantified with the Mithras LB940 instrument with no wavelength filter between the cells and the photomultiplier.

### Bioluminescent Resonance Energy Transfer

BRET was performed as previously described [30] with some minor modifications. GRK2-YFP or β-arrestin 1-YFP replaced β-arrestin 2-YFP, where indicated. Untagged GRK2 was overexpressed at a ratio of 2-fold higher than receptor, while β-arrestin 2-YFP was always kept at the maximum allowable expression. RLuc-tagged D_2_R constructs were not different from untagged receptors in ligand binding and G protein coupling as previously determined for ^[WT]^D_2_R [30] and confirmed for each mutant. Receptor expression levels were determined for each experiment in order to ensure comparable levels of expression.

### Alternative G protein signaling

Various D_2_R constructs expressed in HEK 293 cells were used to determine whether D_2_R could mediate G_αs_ activation in the GloSensor assay described above. cAMP production following stimulation of endogenous β2ARs was used as a control. Whether the various D2R constructs could couple to G_αq_ was measured using the aequorin assay, as previously described [31]. As a control, HEK 293 cells were transfected with the angiotensin AT1A receptor and calcium was measured in response to agonist activation.

### Radioligand Binding

[3H]-raclopride (Promega, Waltman, MA) binding was carried out as previously described [32]. When sodium was removed, the salt was not replaced with any other ion in the buffer. Rluc counts were conducted on the same membrane preparations, the same day that the ligand binding was carried out, using the same RLuc counting protocol as [30].

### Data Analysis

**Fig 1:**
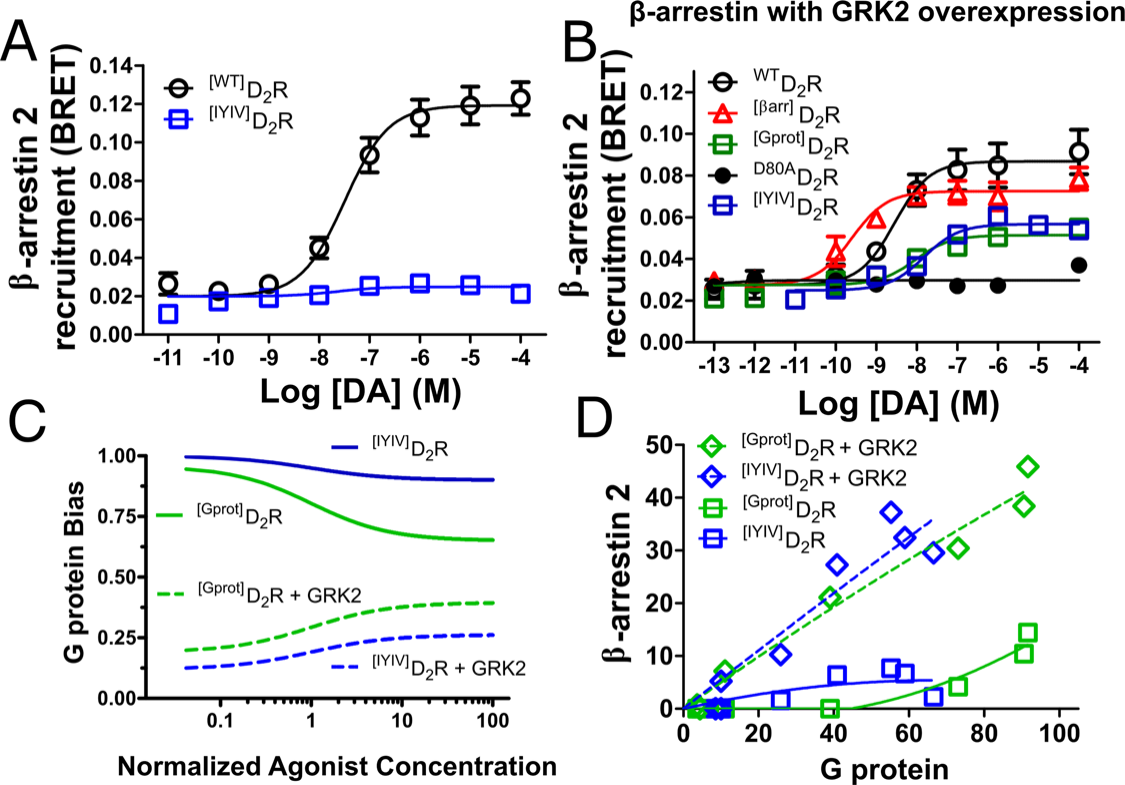
Context dependent functional selectivity. (**A**) β-arrestin 2 recruitment comparing ^[WT]^D_2_R and ^[IYIV]^D_2_R as determined by bioluminescent resonance energy transfer (BRET). (**B**) GRK2 overexpression enhances β-arrestin 2 recruitment by BRET for ^[IYIV]^D_2_R and ^[WT]^D_2_R, but only slightly for ^[Gprot]^D_2_R, ^[βarr]^D_2_R, and ^[D80A]^D_2_R when compared to [28]. All data are presented with SEM from n = 3–4 independent experiments, with statistical significance calculated in S1 Table. Quantification of bias between G protein activity (data presented in S1 Fig and[28]) and β-arrestin 2 recruitment (data presented in Fig 1A and [28]) using (**C**) a statistical formalism where K_A_, calculated from EC_50_ = 1 [33] or (**D**) bias plot mapping under normal (solid lines) and GRK2 overexpression enhanced (broken lines) conditions.

Dose response curves were fit to the nonlinear regression curve y = Bottom + (Top-Bottom)/(1+10^((LogEC_50_-X))) for agonist curves and y = Bottom + (Top-Bottom)/(1+10^((X-LogIC_50_))) for antagonists in Graphpad Prism 5. Statistical tests were performed (described in Table legends) in Graphpad Prism 5. Bias quantification was carried out as previously described in [33] and [34]. For bias plots (Fig 1D and S1C and S1D Fig) the points were calculated from normalized (to ^[WT]^D_2_R) responses to the two assays and fit to a quadratic equation with the constraint that B0 = 0. Each bias quantification used the same G protein activity when compared to endogenous and GRK2 overexpression data sets at β-arrestin. All values calculated in S1–S4 Tables were normalized to ^[WT]^D_2_R (or control receptors in S4 Table) for each individual assay for efficacy but not potency.

## Results

### A rich landscape of receptor dictated functional selectivity

The functional selectivity of a receptor can be simply viewed as its propensity to engage one signaling mechanism over another. The ability of the GPCR kinase 2 (GRK2) to affect the interaction of various D2R mutants with β-arrestin 2 was compared to a previously characterized biased D_2_R that was engineered [35,36] by mutating a motif unique to D2-like receptors (IYIV) to four alanines (^[IYIV]^D_2_R). As seen in Fig 1A and S1A Fig, when expressed in HEK293 cells ^[IYIV]^D_2_R displays a decrease in G protein signaling activity, along with a nearly complete absence of β-arrestin 2 recruitment, as previously observed [36]. Thus, ^[IYIV]^D_2_R is a G protein-preferring mutant receptor under these conditions. However, when GRK2 is overexpressed (Fig 1B), β-arrestin 2 recruitment potency is enhanced at ^[WT]^D_2_R and ^[IYIV]^D_2_R, and only slightly potentiated for the previously characterized ^[Gprot]^D_2_R and ^[βarr]^D_2_R when compared to HEK293 cells expressing endogenous levels of GRK2 [28]. Quantifying the bias between ^[Gprot]^D_2_R and ^[IYIV]^D_2_R using a statistical formalism [33] (Fig 1C and S1B Fig), a bias plot [34] (Fig 1D and S1C and S1D Fig) and ΔΔlog(τ/K_A_) calculations (S2 Table) reveal the quality of G protein bias. These different bias quantifications allow for comparisons of efficacy (calculated from E_MAX_ to τ) and potency (calculated from EC_50_ to K_A_) in different ways, which facilitates conclusions based on their relationship. For instance, ^[Gprot]^D_2_R and ^[IYIV]^D_2_R display similar degrees of bias using each method because while G protein activity is slightly perturbed at ^[IYIV]^D_2_R, at ^[Gprot]^D_2_R the G protein activity is completely intact, while a small amount of β-arrestin activity remains. However, when GRK2 is overexpressed ^[Gprot]^D_2_R does not gain appreciable β-arrestin efficacy, whereas ^[IYIV]^D_2_R gains significantly in efficacy, this is revealed by the greater shift observed using the bias statistical formalism (Fig 1C). In contrast, the bias plot (Fig 1C) reveals that ^[IYIV]^D_2_R is biased toward G protein activity but the mutant never reaches 100% activity, and ΔΔlog(τ/K_A_) values reveal that ^[IVIV]^D_2_R bias is dependent on GRK2 levels (S2 Table). Since the GRK2 overexpression assay demonstrates the receptor’s capacity for β-arrestin recruitment under the most favorable conditions, it shows that ^[IYIV]^D_2_R can display partial agonism at *both* G protein and β-arrestin activities. In contrast, ^[Gprot]^D_2_R retains its original biased signaling profile: full agonism at the G protein pathway and weak partial agonism at the β-arrestin pathway even when GRK2 is highly expressed.

**Fig 2:**
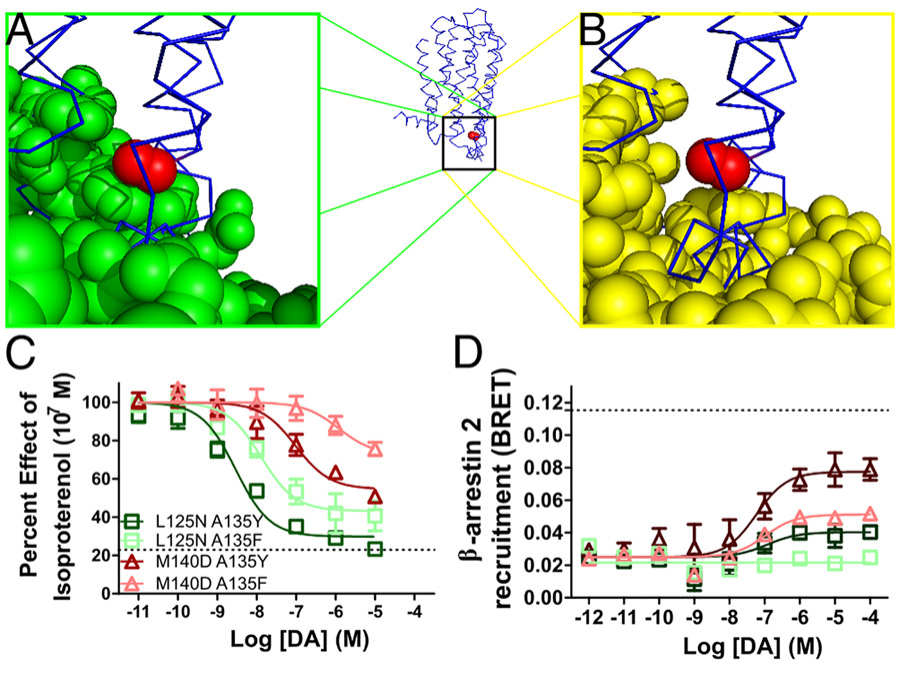
Receptor control of partial agonism at D_2_R with A135 mutations. (**A**) Relative proximity of G proteins (green spheres) and A135 (red sphere) in D3 (blue ribbon, PDB ID: 3PBL [37]) as determined by alignment of D3R to β2AR in receptor/G protein complex (PDB ID: 3SN6, [38]). (**B**) Arrestin (yellow spheres) does not reside close to A135 when D3R is aligned to rhodopsin in receptor/arrestin complex (PDB ID: 4ZWJ [39]). (**C**) G protein activity as determined by inhibition of isoproterenol-induced cAMP accumulation is titrated by substitution of A135 with a bulky polar (tyrosine) or nonpolar (phenylalanine) residue and combined with L125N or M140D to impart controlled loss of G protein function. (**C**) β-arrestin 2 recruitment as determined by BRET is similarly controlled. All data are presented with SEM from n = 3–5 independent experiments, with statistical significance calculated in S1 Table.

The controlled perturbation of G protein and β-arrestin pathways allows for a more detailed examination of partial agonism and how it can be utilized in generating receptor bias. The conserved residue A3.53 (A135) was previously mutated into all 19 possible amino acids [28] because it conferred remarkable functional selectivity properties. In fact, when the proximity of A135 to the G protein in the receptor/G protein complex (Fig 2A) is compared to its proximity to arrestin in the receptor/arrestin complex (Fig 2B) it is clear that the G protein more closely associates with A135. When A135 is mutated to basic residues, D_2_R lost ~50% of its G protein activity, while still retaining complete, and slightly more potent β-arrestin activity (S2A and S2B Fig, respectively). In contrast, acidic substitutions ablated activity at both pathways. Furthermore, substitution with a bulky polar residue (tyrosine) yielded a balanced reduction in both G protein and β-arrestin 2 activity to roughly 75% (S2C and S2D Fig) and substitution with a bulky nonpolar residue (phenylalanine) yielded a balanced 50% reduction (S2C and S2D Fig). These mutants were combined with one residue substitution from ^[Gprot]^D_2_R (L125N) or ^[βarr]^D_2_R (M140D) to generate mutants that maintain the loss of signaling associated with either ^[Gprot]^D_2_R or ^[βarr]^D_2_R while also exhibiting partial agonism at the retained pathway. In other words, these mutants selectively lose signaling at on pathway nearly completely, while allowing precise control of the degree of efficacy (partial agonism) still present at the other pathway.

### Agonist texture reveals novel modes of functional selectivity

**Fig 3:**
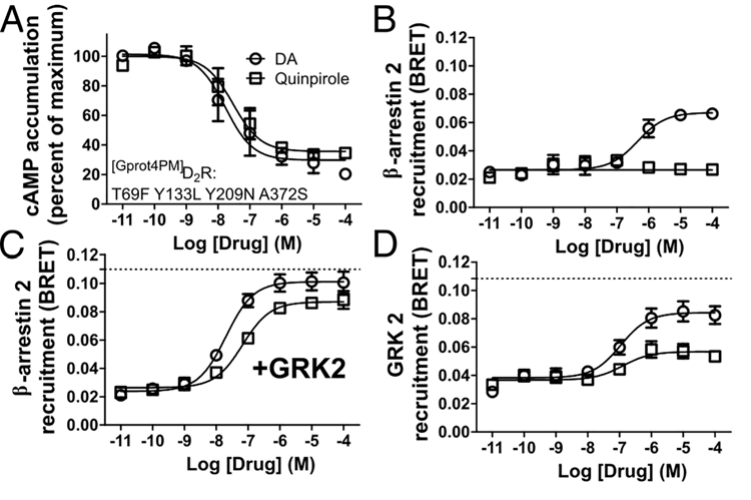
A unique G protein biased mutant demonstrates agonist texture. (**A**) Dopamine (DA) and quinpirole equivalently inhibit cAMP production, which is equivalent to ^[WT]^D_2_R for ^[Gprot4PM]^D_2_R (T69F Y133L Y209N A372S). (**B**) ^[Gprot4PM]^D_2_R has roughly 50% efficacy in response to DA but not quinpirole for β-arrestin 2 recruitment. (**C**) GRK2 overexpression rescues both DA and quinpirole β-arrestin 2 recruitment activity nearly to ^[WT]^D_2_R levels (dotted line, from Fig 1B). (**D**) GRK2 recruitment as determined by BRET (where GRK2 is tagged with YFP) shows the same ligand discrepancy as β-arrestin 2. All data are presented with SEM from n = 3 independent experiments, with statistical significance calculated in S3 Table.

A unique G protein-biased mutant (termed ^[Gprot4PM]^D_2_R) displayed remarkable retention of G protein activity and loss of β-arrestin 2 recruitment when the reference agonist quinpirole was used to probe activity (Fig 3A and 3B, respectively). This mutation was generated by the same iterative Evolutionary Trace process that produced ^[Gprot]^D_2_R and ^[βarr]^D_2_R [28]. However, when the endogenous ligand DA was used, the G protein activity was unchanged, but the β-arrestin activity was ~50% of ^[WT]^D_2_R (Fig 3A and 3B, S3 Table). At ^[Gprot4PM]^D_2_R both DA and quinpirole were able to recruit β-arrestin 2 to almost the same extent of ^[WT]^D_2_R when GRK2 was overexpressed (Fig 3C, S3 Table). However, the recruitment of GRK2 by ^[Gprot4PM]^D_2_R showed the same agonist selectivity between DA and quinpirole (Fig 3D) as observed with β-arrestin 2. These data demonstrate the concept of *agonist texture* [40], which predicts that full agonists induce distinct receptor activation states and conformations.

**Fig 4:**
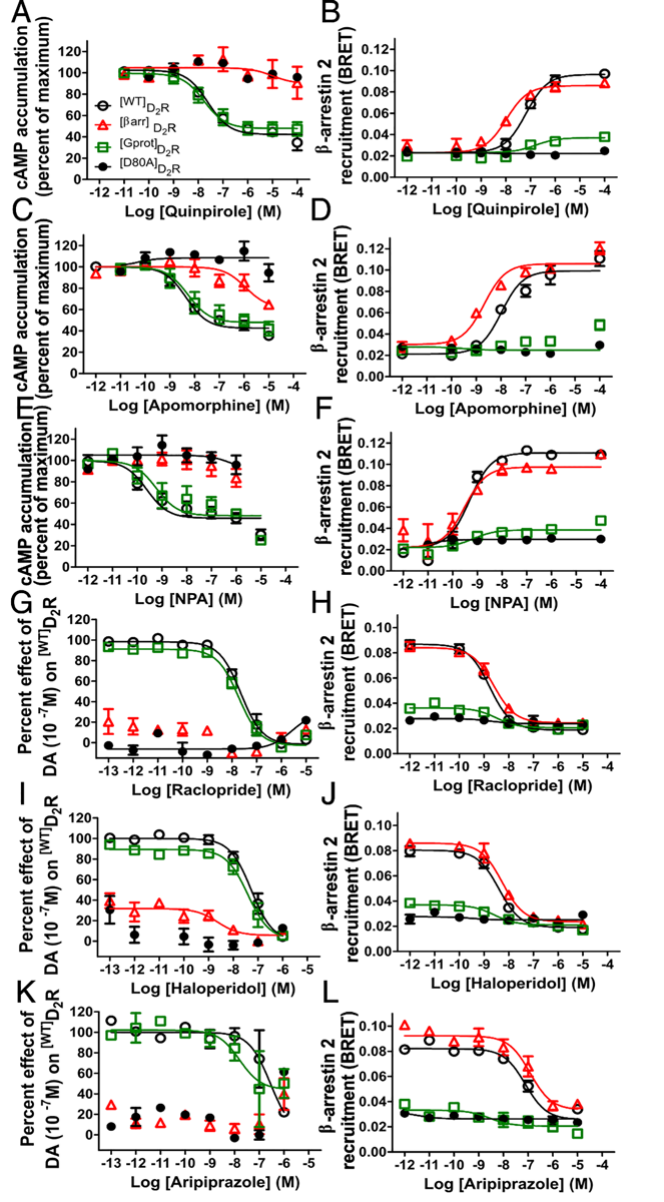
Agonists and antagonists with diverse pharmacophores elicit predictable responses at ^[Gprot]^D_2_R and ^[βarr]^D_2_R. The D_2_R agonists quinpirole, apomorphine, and N-propylapomorphine (NPA) were tested for G protein activity (**A,C,E**) and β-arrestin 2 recruitment (**B,D,F**). For each agonist, ^[Gprot]^D_2_R showed a response similar to ^[WT]^D_2_R at G protein activation and more similar to ^[D80A]^D_2_R for β-arrestin recruitment, while ^[βarr]^D_2_R was not active at the G protein pathway but retained activity at the β-arrestin pathway. The antagonists raclopride (**G,H**) haloperidol (**I,J**) and partial antagonist aripiprazole (**K,L**) were able to block DA elicited D_2_R activation at the G protein pathway (**G,I,K**) for ^[Gprot]^D_2_R and ^[WT]^D_2_R to the same extent, while ^[D80A]^D_2_R and ^[βarr]^D_2_R had no effect to inhibit. In contrast, these antagonists block DA elicited β-arrestin 2 recruitment (**H,J,L**) for ^[βarr]^D_2_R and ^[WT]^D_2_R. All data are presented with SEM from n = 3–4 independent experiments, with statistical significance calculated in S3 Table.

While ^[Gprot]^D_2_R and ^[βarr]^D_2_R display unprecedented separation of signal in response to DA [28], various additional D_2_R agonists and antagonists were used to probe the extent of agonist texture between the mutants at G protein and β-arrestin 2 activation. Each agonist tested at cAMP inhibition was effectively equivalent for ^[WT]^D_2_R and ^[Gprot]^D_2_R while being severely disrupted for ^[βarr]^D_2_R and ^[D80A]^D_2_R (Fig 4A, 4C and 4E, S3 Table). In contrast, ^[βarr]^D_2_R was nearly as effective at β-arrestin 2 recruitment as ^[WT]^D_2_R whereas ^[Gprot]^D_2_R and ^[D80A]^D_2_R were deficient (Fig 4B, 4D and 4F, S3 Table). Similarly, the well characterized antagonist raclopride, typical antipsychotic haloperidol, and atypical antipsychotic aripiprazole inhibited DA-induced cAMP reduction for ^[Gprot]^D_2_R and ^[WT]^D_2_R (Fig 4G, 4I and 4K, S3 Table), and β-arrestin 2 recruitment for ^[βarr]^D_2_R and ^[WT]^D_2_R (Fig 4H, 4J and 4L, S3 Table). These diverse D_2_R ligands behave as expected for each assay and provide evidence that the complex activation states of ^[Gprot]^D_2_R and ^[βarr]^D_2_R remain intact, as opposed to ^[Gprot4PM]^D_2_R, which has lost responsiveness to quinpirole at β-arrestin 2 recruitment selectively.

### The status of receptor interacting partners in extremely biased mutant D_2_Rs

**Fig 5:**
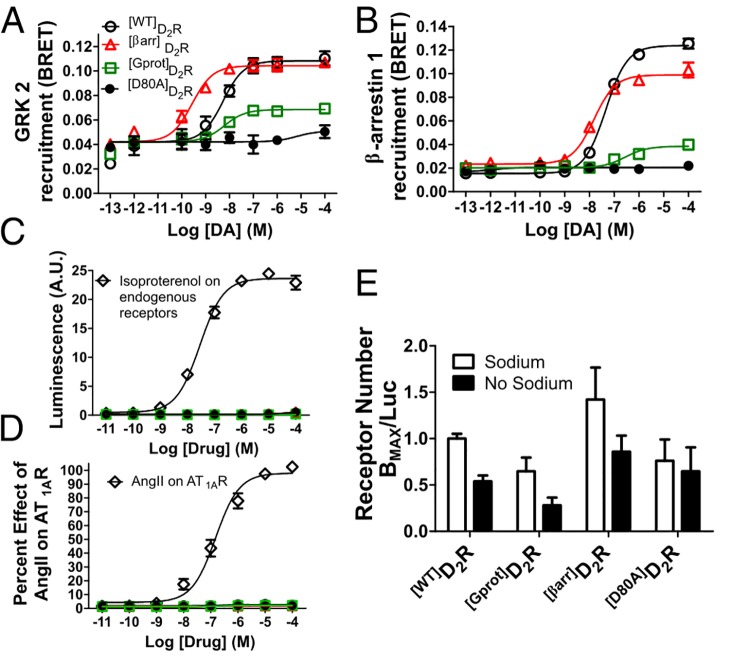
Interacting partners and allosteric D_2_R determinants of functional selectivity. (**A**) GRK2 and (**B**) β-arrestin 1 recruitment as assessed by BRET show a similar profile as β-arrestin 2: ^[βarr]^D_2_R recruits normally, while ^[Gprot]^D_2_R is severely deficient. (**C**) Each D_2_R construct was expressed in HEK 293T cells and assessed for its ability to stimulate cAMP in response to DA. Stimulation of endogenous receptor by isoproterenol was used as a control response. (**D**) G_αq_ mediated Ca^2+^ flux, as measured by the aequorin luminescence assay, is not stimulated by ^[WT]^D_2_R, ^[Gprot]^D_2_R, ^[βarr]^D_2_R or ^[D80A]^D_2_R, compared to AngII induced Ca^2+^ flux induced by transient expression of AT_1A_R. (**E**) B_MAX_ was determined by binding, while luciferase-tagged receptors provided a B_MAX_-independent measure of receptor number. In this assay, the responsiveness to sodium is retained for all mutants (except ^[D80A]^D_2_R). All data are presented with SEM from n = 3–4 independent experiments, with statistical significance calculated in S4 Table.

Allosteric modulators were assessed at ^[Gprot]^D_2_R and ^[βarr]^D_2_R while being compared to positive and negative controls (^[WT]^D_2_R and ^[D80A]^D_2_R, respectively). The interaction of each receptor with other components of the desensitization machinery mirrored the recruitment of β-arrestin 2. GRK2 (Fig 5A) and β-arrestin 1 (Fig 5B) showed a similar slight potentiation and loss of efficacy at ^[βarr]^D_2_R when compared to ^[WT]^D_2_R, while ^[Gprot]^D_2_R was severely deficient (S4 Table). To test whether the mutagenesis mediated loss of function at G protein and β-arrestin interactions achieved with ^[Gprot]^D_2_R and ^[βarr]^D_2_R could potentially have induced aberrant activation of normally inactive receptor interacting proteins, two other non-conventional D_2_R signaling avenues (G_αs_ and G_αq_) were assessed and neither were activated by any of the D_2_R mutants (Fig 5C and 5D, respectively, S4 Table).

The effect of the GPCR allosteric modulator sodium has been previously shown to enhance dopamine binding to D_2_R [29] and has been proposed to function as an efficacy switch for receptor bias [41]. As previously demonstrated [29], when sodium is removed from D_2_R binding buffer this results in a 50% reduction in B_MAX_, similar to the phenomenon observed in the A_2A_R [42]. To assess sodium dynamics in D_2_R, radioligand binding was used to determine B_MAX_ with and without sodium on the *Renilla luciferase* tagged D_2_R constructs [30]. In this way, luminescence counts yield total receptors available for binding (relative to ^[WT]^D_2_R) and B^MAX^ yields total binding sites, modulated by sodium. This assay yielded a 50% reduction in ^[WT]^D_2_R and ^[βarr]^D_2_R apparent B_MAX_ (Fig 5E) when binding was performed without sodium and ^[Gprot]^D_2_R also showed a sodium dependent reduction, although ^[Gprot]^D_2_R expresses lower than ^[WT]^D_2_R as previously described [28]. ^[D80A]^D_2_R B_MAX_ did not change regardless of the presence of sodium, which validates the experiment as ^[D80A]^D_2_R is mutated at the presumed site of sodium interaction and has been previously shown to not bind sodium [29].

## Discussion

The functional selectivity of D_2_R is dynamic and malleable. Ligands that target D_2_R have significant therapeutic impact [24] and the quality of agonism or antagonism of these ligands can be operationally defined in monitoring systems [8,30]. The agonist or antagonist quality of a ligand/receptor pair is dependent upon the assay used for detection [43]. Here, the capacity, quality and character of D_2_R functional selectivity has been examined for different mutated D_2_Rs.

GPCR bias is operationally defined by the degree of engagement of a given signaling pathway versus another. It is clear that ^[IYIV]^D_2_R and ^[Gprot]^D_2_R both share G protein bias, however the quality of bias differs, depending on their operational definition. For example, in some cases it may be beneficial to retain all of the G protein activity, while in others it may be crucial to abolish all β-arrestin recruitment at the sacrifice of some G protein activity. In fact, ^[IYIV]^D_2_R was mutated to contain fewer alanine substitutions which resulted in full G protein activity and partial β-arrestin activity [36]. Nevertheless, both ^[IYIV]^D_2_R and ^[Gprot]^D_2_R were used to dissect D_2_R-mediated ERK phosphorylation [28,35] and both mutants yielded a similar conclusion: that D_2_R-mediated ERK phosphorylation is largely G protein mediated.

Operational consistency allows for meaningful conclusions about D_2_R signaling pathways to be drawn. The partial agonist activity of aripiprazole has raised the possibility that complete blockade of D_2_R is not necessary to impart antipsychotic efficacy [44]. This partial agonism allows for effective targeting of G protein or β-arrestin pathways [8]. However, the causal relationship of partial agonism and biased partial agonism (as opposed to partial antagonism) has not been explored. The mutants described here that are derivations of A135-mutated D_2_R are tools that would allow for operationally defined agonism.

While receptor manipulation is desirable to demonstrate causal relationships, biased ligands provide valuable insight and are a more reasonable avenue toward therapy development. However, precise details and principles governing ligand action remain elusive. Here, the phenomenon of agonist texture [40] is demonstrated with ^[Gprot4PM]^D_2_R. As previously observed, full agonists can stabilize different receptor activation states that have functional consequences [40]. Therefore, functional selectivity could occur from the loss of function at one pathway or it could be thought of as a gain of new receptor activity. This phenomenon represents a valuable conceptual framework for a fundamental property of receptor activation that is relevant to functional selectivity.

Intracellular signal transduction proteins are key elements in dictating bias. Their interactions with receptors dictate agonist efficacy [45] and targeting their activation with biased agonists is an avenue by which already validated receptor targets can be leveraged for improved therapies. Here, the related desensitization allosteric modulators (β-arrestin 1, β-arrestin 2, and GRK2, Fig 5A and 5B) were shown to fall into a common activation family using ^[βarr]^D_2_R. These findings could have an impact on future studies of more detailed elements of D_2_R’s β-arrestin signaling arm, such as barcoded phosphorylation patterns [46], GRK subfamily contributions [47], pleiotropic β-arrestin conformation states [48], and other posttranslational modifications [49]. Additionally, related allosteric modulators (different G proteins, Fig 5C and 5D) remained inactive at each mutant receptor, which indicates that none of the mutants have a gross abnormal gain of function.

Interactions with small molecule allosteric modulators are also exciting avenues by which functional selectivity can be modulated. Sodium, an intracellular GPCR allosteric modulator, binds both ^[Gprot]^D_2_R and ^[βarr]^D_2_R, indicating that both G protein and β-arrestin activation require dynamic sodium regulation. However, allosteric biased ligands may confer functional selectivity by exploiting the recently solved extracellular vestibule [50] to generate noncompetitive negative or positive allosteric modulators [51] or bitopic ligands [52].

Novel mutants and rigorous examinations of strongly biased mutants provided a conceptual framework for the feasibility of such exercises. Furthermore, these studies provide robust and versatile tools for further investigations of partial agonism or agonist texture in complex, physiologically relevant systems. The actions of dopamine, and D_2_R, are dysregulated in many neurological and psychiatric disorders, yet the complete and precise molecular actions of D_2_R remain elusive. The work presented here highlights new tools available for the molecular dissection of D_2_R and provides valuable insight into methods that can be used to increase the utility of targeting D_2_R pharmacologically for improved therapeutics.

## Supporting Information

S1 Fig Comparison of receptor bias. (**A**) cAMP partial agonism at ^[IYIV]^D_2_R recapitulates previously published values [36]. Data are presented with SEM from n = 3 independent experiments. (**B**) Comparison of each biased mutant quantified using a statistical formalism [33] with endogenous GRK levels (solid lines) compared to GRK2 overexpression (broken lines). (**C**) and (**D**) bias plots to compare each receptor with and without GRK2 overexpressed, respectively. The data presented in B,C, and D is the full data set of mutants, while Fig 1C and 1D show only the G protein-biased mutants of these data.
(TIF)

S2 Fig Molecular determinants of signal efficacy at A135. (**A**) G protein activity, as assessed by cAMP inhibition and (**B**) β-arrestin 2 recruitment, as assessed by BRET are compared to ^[WT]^D_2_R efficacy for the G protein pathway (dotted line, A) and potency for the β-arrestin 2 recruitment (dotted line, B) respectively. Basic residue substitutions (blue) strongly bias D_2_R toward β-arrestin with an increase in potency, while acidic residues ablate signaling at both pathways. All data are presented with SEM from n = 3 independent experiments.
(TIF)

S1 Table Receptor controlled perturbation of functional selectivity. Values derived from Figs 1 and 2 to demonstrate the receptor’s contributions to functional selectivity. *p<0.05 when compared to ^[WT]^D_2_R for efficacy and potency as determined by Bonferroni post-hoc test after p<0.05 for one-way ANOVA.
(DOCX)

S2 Table Quantifying bias at G protein preferring mutant D_2_R. B_INF_ and ΔΔlog(τ/KA) were calculated according to references in the table. Some control data (DA at cAMP inhibition and β-arrestin 2 recruitment for ^[WT]^D_2_R) calculated from [28].
(DOCX)

S3 Table Ligand contributions to functional selectivity. Calculated from Figs 3 and 4. *p<0.05 when compared to ^[WT]^D_2_R for efficacy and potency at each ligand as determined by Bonferroni post-hoc test after p<0.05 by one-way ANOVA.
(DOCX)

S4 Table Transducer contributions to functional selectivity. Calculated from Fig 5. *p<0.05 when compared to ^[WT]^D_2_R or control receptors (β_2A_R for G_αs_ or AT_1A_R for G_αq_) for efficacy and potency as determined by Bonferroni post-hoc test after p<0.05 for one-way ANOVA.
(DOCX)
