# Supplementary figures and images for: Receptor, Ligand and Transducer Contributions to Dopamine D2 Receptor Functional Selectivity
Source: PLoS One. 2015 Oct 30;10(10):e0141637. doi: 10.1371/journal.pone.0141637 (PMC4627803; doi:10.1371/journal.pone.0141637)

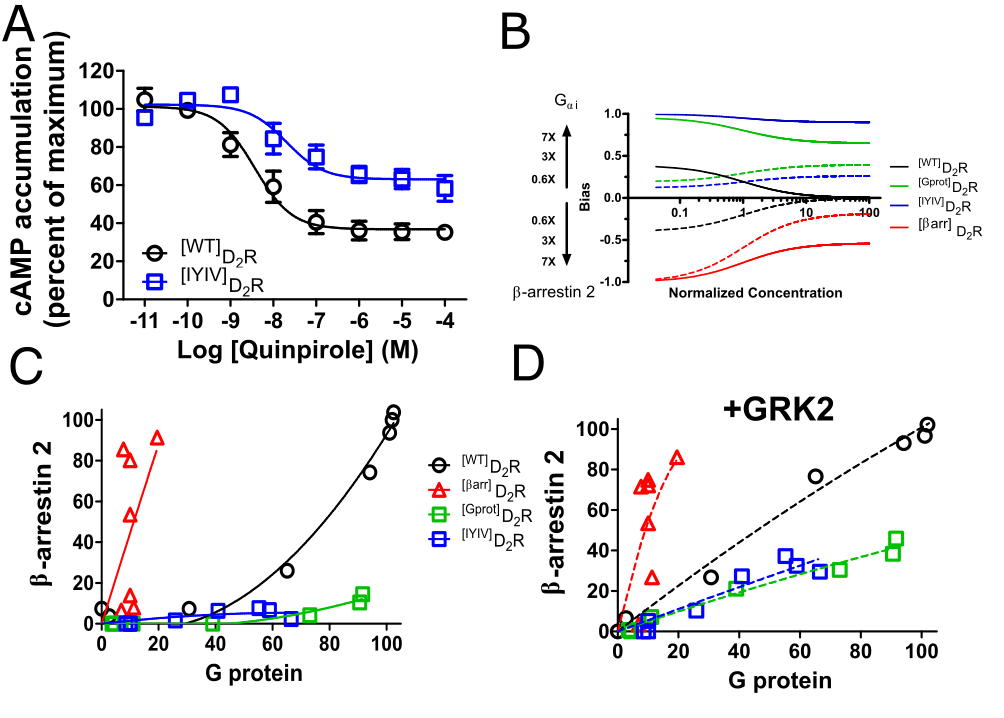

Supplement: S1 Fig — (A) cAMP partial agonism at [IYIV]D2R recapitulates previously published values [36]. Data are presented with SEM from n = 3 independent experiments. (B) Comparison of each biased mutant quantified using a statistical formalism [33] with endogenous GRK levels (solid lines) compared to GRK2 overexpression (broken lines). (C) and (D) bias plots to compare each receptor with and without GRK2 overexpressed, respectively. The data presented in B,C, and D is the full data set of mutants, while Fig 1C and 1D show only the G protein-biased mutants of these data. (TIF) [file pone.0141637.s001.tif]

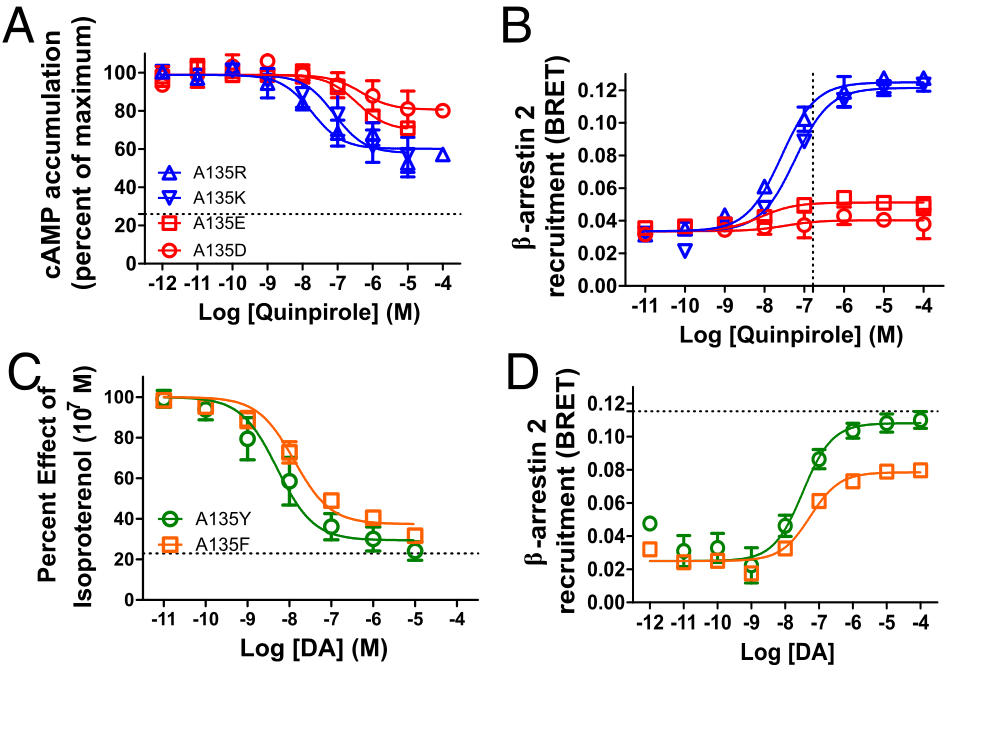

Supplement: S2 Fig — (A) G protein activity, as assessed by cAMP inhibition and (B) β-arrestin 2 recruitment, as assessed by BRET are compared to [WT]D2R efficacy for the G protein pathway (dotted line, A) and potency for the β-arrestin 2 recruitment (dotted line, B) respectively. Basic residue substitutions (blue) strongly bias D2R toward β-arrestin with an increase in potency, while acidic residues ablate signaling at both pathways. All data are presented with SEM from n = 3 independent experiments. (TIF) [file pone.0141637.s002.tif]
