# Supplementary material for: Receptor, Ligand and Transducer Contributions to Dopamine D2 Receptor Functional Selectivity
Source: PLoS One. 2015 Oct 30;10(10):e0141637. doi: 10.1371/journal.pone.0141637 (PMC4627803; doi:10.1371/journal.pone.0141637)
Supplement: S1 Table — Values derived from Figs 1 and 2 to demonstrate the receptor’s contributions to functional selectivity. *p<0.05 when compared to [WT]D2R for efficacy and potency as determined by Bonferroni post-hoc test after p<0.05 for one-way ANOVA. (DOCX) [file pone.0141637.s003.docx]

| Mutant | Assay (ligand) | EC_50_ (nM) | E_MAX_ (% ^[WT]^D_2_R) | Figure |
| --- | --- | --- | --- | --- |
| ^[IYIV]^D_2_R | β-arrestin 2 (DA) | 0.2 +/- 0.3* | 13 +/- 3* | 1A |
|  | β-arrestin 2+GRK2 (DA) | 15 +/- 2* | 36 +/- 3* | 1B |
| ^[Gprot]^D_2_R | β-arrestin 2+GRK2 (DA) | 5.3 +/- 1.8 | 44 +/- 5* | 1B |
| A135Y | cAMP (DA) | 7 +/- 2 | 85 +/- 8 | S2C |
|  | β-arrestin 2 (DA) | 33 +/- 1 | 79 +/- 4 | S2D |
| A135Y L125N | cAMP (DA) | 6 +/- 1 | 81 +/- 4 | 2C |
|  | β-arrestin 2 (DA) | 120 +/- 10* | 15 +/- 5* | 2D |
| A135Y M140D | cAMP (DA) | 110 +/- 2* | 57 +/- 6* | 2C |
|  | β-arrestin 2 (DA) | 59 +/- 2* | 50 +/- 5* | 2D |
| A135F | cAMP (DA) | 18 +/- 1* | 75 +/- 4 | S2C |
|  | β-arrestin 2 (DA) | 55 +/- 2* | 51 +/- 4* | S2D |
| A135F L125N | cAMP (DA) | 16 +/- 2* | 69 +/- 6 | 2C |
|  | β-arrestin 2 (DA) | 440 +/- 2500* | 3.1 +/- 5.9* | 2D |
| A135F M140D | cAMP (DA) | 580 +/- 3* | 38 +/- 10* | 2C |
|  | β-arrestin 2 (DA) | 69 +/- 3* | 29 +/- 5* | 2D |
|  |  |  |  |  |
|  |  |  |  |  |
