# Supplementary material for: Receptor, Ligand and Transducer Contributions to Dopamine D2 Receptor Functional Selectivity
Source: PLoS One. 2015 Oct 30;10(10):e0141637. doi: 10.1371/journal.pone.0141637 (PMC4627803; doi:10.1371/journal.pone.0141637)
Supplement: S2 Table — BINF and ΔΔlog(τ/KA) were calculated according to references in the table. Some control data (DA at cAMP inhibition and β-arrestin 2 recruitment for [WT]D2R) calculated from [28]. (DOCX) [file pone.0141637.s004.docx]

| Mutant | Calculation Method | Bias Factor | Bias Factor with GRK2 | Figure |
| --- | --- | --- | --- | --- |
| ^[IYIV]^D_2_R | Barak and Peterson, 2012 | 0.9 (B_INF_) | 0.3 (B_INF_) | 1C |
| ^[Gprot]^D_2_R | β-arrestin 2+GRK2 (DA) | 0.7 (B_INF_) | 0.4 (B_INF_) | 1C |
| ^[IYIV]^D_2_R | Kenakin and Christopoulos, 2013 | 1.5e4 (10^ΔΔlog(τ/K_A_)) | 3.2 (10^ΔΔlog(τ/K_A_)) | 1D |
| ^[Gprot]^D_2_R |  | 20 (10^ΔΔlog(τ/K_A_)) | 3.2 (10^ΔΔlog(τ/K_A_)) | 1D |
|  |  |  |  |  |
|  |  |  |  |  |
|  |  |  |  |  |
|  |  |  |  |  |
|  |  |  |  |  |
|  |  |  |  |  |
|  |  |  |  |  |
|  |  |  |  |  |
|  |  |  |  |  |
|  |  |  |  |  |
|  |  |  |  |  |
|  |  |  |  |  |
|  |  |  |  |  |
|  |  |  |  |  |
|  |  |  |  |  |
|  |  |  |  |  |
|  |  |  |  |  |
|  |  |  |  |  |
|  |  |  |  |  |
