# Supplementary material for: Receptor, Ligand and Transducer Contributions to Dopamine D2 Receptor Functional Selectivity
Source: PLoS One. 2015 Oct 30;10(10):e0141637. doi: 10.1371/journal.pone.0141637 (PMC4627803; doi:10.1371/journal.pone.0141637)
Supplement: S3 Table — Calculated from Figs 3 and 4. *p<0.05 when compared to [WT]D2R for efficacy and potency at each ligand as determined by Bonferroni post-hoc test after p<0.05 by one-way ANOVA. (DOCX) [file pone.0141637.s005.docx]

| Mutant | Ligand (Assay) | EC_50/_IC_50_ (nM) | E_MAX/_IC_MAX_ (% ^[WT]^D_2_R) | Figure |
| --- | --- | --- | --- | --- |
| ^[Gprot4PM]^D_2_R | DA (cAMP) | 17 ± 2* | 100 ± 11 | 3A |
|  | Quinpirole (cAMP) | 29 ± 2 | 89 ± 7 | 3A |
|  | DA (β-arrestin 2) | 450 ± 20* | 48 ± 4* | 3B |
|  | Quinpirole (β-arrestin 2) | Ambiguous Fit | | 3B |
|  | DA (β-arrestin 2 + GRK2) | 20 ± 1* | 89 ± 4 | 3C |
|  | Quinpirole (β-arrestin2+GRK2) | 68 ± 1* | 69 ± 3* | 3C |
|  | DA (GRK2) | 110 ± 2* | 63 ± 6* | 3D |
|  | Quinpirole (GRK2) | 130 ± 2* | 27 ± 4* | 3D |
| ^[WT]^D_2_R | Quinpirole (cAMP) | 25 ± 1 | 100 ± 6 | 4A |
|  | Apomorphine (cAMP) | 4.6 ± 1 | 100 ± 6 | 4C |
|  | NPA (cAMP) | 0.2 ± 0.1 | 98 ± 11 | 4E |
| ^[Gprot]^D_2_R | Quinpirole (cAMP) | 17 ± 2* | 86 ± 8 | 4A |
|  | Apomorphine (cAMP) | 5.6 ± 2 | 91 ± 11 | 4C |
|  | NPA (cAMP) | 0.6 ± 0.1 | 94 ± 14 | 4E |
| ^[βarr]^D_2_R | Quinpirole (cAMP) | 10,000 ± 2,500* | 25 ± 20* | 4A |
|  | Apomorphine (cAMP) | 1,300 ± 229* | 67 ± 15 | 4C |
|  | NPA (cAMP) | Not Converged | | 4E |
| ^[D80A]^D_2_R | Quinpirole (cAMP) | 510 ± 400* | 14 ± 10* | 4A |
|  | Apomorphine (cAMP) | Ambiguous Fit | | 4C |
|  | NPA (cAMP) | 650 ± 320* | 27 ± 90 | 4E |
| ^[WT]^D_2_R | Quinpirole (β-arrestin 2) | 66 ± 1 | 100 ± 4 | 4B |
|  | Apomorphine (β-arrestin 2) | 10 ± 1 | 100 ± 7 | 4D |
|  | NPA (β-arrestin 2) | 0.5 ± 0.1 | 100 ± 4 | 4F |
| ^[Gprot]^D_2_R | Quinpirole (β-arrestin 2) | 160 ± 28 | 19 ± 4* | 4B |
|  | Apomorphine (β-arrestin 2) | 1,700 ± 200* | 29 ± 5* | 4D |
|  | NPA (β-arrestin 2) | 0.7 ± 0.4 | 19 ± 4* | 4F |
| ^[βarr]^D_2_R | Quinpirole (β-arrestin 2) | 11 ± 1 | 86 ± 3 | 4B |
|  | Apomorphine (β-arrestin 2) | 2.0 ± 0.2 | 97 ± 6 | 4D |
|  | NPA (β-arrestin 2) | 0.3 ± 0.1 | 86 ± 4 | 4F |
| ^[D80A]^D_2_R | Quinpirole (β-arrestin 2) | Ambiguous Fit | | 4B |
|  | Apomorphine (β-arrestin 2) | Ambiguous Fit | | 4D |
|  | NPA (β-arrestin 2) | 0.1 ± 0.8 | 9 ± 4* | 4F |
| ^[WT]^D_2_R | Raclopride (cAMP) | 23 ± 1 | 100 ± 4 | 4G |
|  | Haloperidol (cAMP) | 54 ± 1 | 100 ± 6 | 4I |
|  | Aripiprazole (cAMP) | 280 ± 23 | 100 ± 25 | 4K |
| ^[Gprot]^D_2_R | Raclopride (cAMP) | 19 ± 1 | 94 ± 4 | 4G |
|  | Haloperidol (cAMP) | 39 ± 1 | 89 ± 6 | 4I |
|  | Aripiprazole (cAMP) | 16 ± 3* | 58 ± 16 | 4K |
| ^[βarr]^D_2_R | Raclopride (cAMP) | Not Converged | | 4G |
|  | Haloperidol (cAMP) | 2.3 ± 2.6* | 26 ± 5* | 4I |
|  | Aripiprazole (cAMP) | Not Converged | | 4K |
| ^[D80A]^D_2_R | Raclopride (cAMP) | Ambiguous Fit | | 4G |
|  | Haloperidol (cAMP) | Not Converged | | 4I |
|  | Aripiprazole (cAMP) | Not Converged | | 4K |
| ^[WT]^D_2_R | Raclopride (β-arrestin 2) | 1.7 ± 1.2 | 100 ± 3 | 4H |
|  | Haloperidol (β-arrestin 2) | 4.0 ± 1.2 | 100 ± 4 | 4J |
|  | Aripiprazole (β-arrestin 2) | 77 ± 2 | 99 ± 7 | 4L |
| ^[Gprot]^D_2_R | Raclopride (β-arrestin 2) | 3.1 ± 2 | 23 ± 3* | 4H |
|  | Haloperidol (β-arrestin 2) | 2.6 ± 1.8 | 26 ± 3* | 4J |
|  | Aripiprazole (β-arrestin 2) | 2.8 ± 4.8* | 22 ± 7* | 4L |
| ^[βarr]^D_2_R | Raclopride (β-arrestin 2) | 2.5 ± 1.2 | 88 ± 4 | 4H |
|  | Haloperidol (β-arrestin 2) | 5.5 ± 1.3 | 100 ± 5 | 4J |
|  | Aripiprazole (β-arrestin 2) | 120 ± 16* | 100 ± 11 | 4L |
| ^[D80A]^D_2_R | Raclopride (β-arrestin 2) | 1.9 ± 13 | 6 ± 3* | 4H |
|  | Haloperidol (β-arrestin 2) | 0.3 ± 1.2 | 4 ± 4* | 4J |
|  | Aripiprazole (β-arrestin 2) | 0.1 ± 0.3* | 10 ± 11* | 4L |
