# Supplementary material for: Receptor, Ligand and Transducer Contributions to Dopamine D2 Receptor Functional Selectivity
Source: PLoS One. 2015 Oct 30;10(10):e0141637. doi: 10.1371/journal.pone.0141637 (PMC4627803; doi:10.1371/journal.pone.0141637)
Supplement: S4 Table — Calculated from Fig 5. *p<0.05 when compared to [WT]D2R or control receptors (β2AR for Gαs or AT1AR for Gαq) for efficacy and potency as determined by Bonferroni post-hoc test after p<0.05 for one-way ANOVA. (DOCX) [file pone.0141637.s006.docx]

| Receptor | Assay (Ligand) | EC_50_ (nM) | E_MAX_ (% Control) | Figure |
| --- | --- | --- | --- | --- |
| ^[WT]^D_2_R | GRK2 recruitment (DA) | 4.6 ± 1.5 | 100 ± 4 | 5A |
| ^[Gprot]^D_2_R |  | 6 ± 2 | 40 ± 4* | 5A |
| ^[βarr]^D_2_R |  | 0.3 ± 0.2 | 94 ± 4 | 5A |
| ^[D80A]^D_2_R |  | 6,600 ± 250* | 14 ± 10* | 5A |
| ^[WT]^D_2_R | β-arrestin 1 recruitment (DA) | 46 ± 1 | 100 ± 2 | 5B |
| ^[Gprot]^D_2_R |  | 280 ± 10* | 17 ± 2* | 5B |
| ^[βarr]^D_2_R |  | 15 ± 1* | 70 ± 2* | 5B |
| ^[D80A]^D_2_R |  | 0.1 ± 1.5* | 3 ± 3* | 5B |
| Endogenous β_2A_R | Gαs (isoproterenol) | 29 ± 1 | 100 ± 2 | 5C |
| ^[WT]^D_2_R | (DA) | 8,800 ± 950* | 1 ± 1* | 5C |
| ^[Gprot]^D_2_R |  | Ambiguous Fit | | 5C |
| ^[βarr]^D_2_R |  | 1.8e8 ± 1.5e7* | 5 ± 7* | 5C |
| ^[D80A]^D_2_R |  | 1.4e8 ± 8.9e7* | 4 ± 6* | 5C |
| AT_1A_R | G_αq_ (ANGII) | 150 ± 12 | 94 ± 3 | 5D |
| ^[WT]^D_2_R | (DA) | 210 ± 40 | 1 ± 1* | 5D |
| ^[Gprot]^D_2_R |  | 270 ± 34 | 1 ± 1* | 5D |
| ^[βarr]^D_2_R |  | 77 ± 52 | 1 ± 1* | 5D |
| ^[D80A]^D_2_R |  | Not Converged | | 5D |
